# Too many cooks could spoil the broth: choice overload and the provision of ambulatory health care

**DOI:** 10.1007/s10754-024-09379-y

**Published:** 2024-05-27

**Authors:** Helmut Herwartz, Christoph Strumann

**Affiliations:** 1 Chair for Econometrics, University of Goettingen, Humboldtallee 3, 37073 Goettingen, Germany; 2 Institute of Family Medicine, University Medical Center Schleswig-Holstein, Campus Luebeck, Ratzeburger Allee 160, 23538 Luebeck, Germany

**Keywords:** Choice overload, Patient choice, Health care coordination, Spatial distribution of health services, C21, C23, D12, D83, I12, I18

## Abstract

Patient empowerment calls for an intensified participation of (informed) patients with more treatment opportunities to choose from. A growing body of literature argues that confronting consumers with too many opportunities can lead to a choice overload (CO) resulting in uncertainty that the selected alternative dominates all other options in the choice set. We examine whether there is a CO effect in the demand for ambulatory health care in Germany by analyzing the association of medical specialists supply on so-called patients’ health uncertainty. Further, we investigate if the CO effect is smaller in areas with a higher density of general practitioners (GPs). We find that patients who live in an area with a large supply of specialists are subject to a CO effect that is expressed by an increased health uncertainty. The coordinating role of GPs seems to be effective to reduce the CO effect, while preserving free consumer choice.

**Supplementary Information:**

The online version contains supplementary material available at 10.1007/s10754-024-09379-y.

## Introduction

A growing body of literature argues that confronting consumers with too many choice alternatives could lead to a choice overload (CO, Chernev et al., 2015; Schwartz, 2004). Potential consequences are a reduced motivation to choose (Maltz & Rachmilevitch, 2021; Frank & Lamiraud, 2009; Iyengar & Lepper, 2000; Tversky & Shafir, 1992), a weakened decision quality (Heiss et al., 2013; Besedeš et al., 2012), dissatisfaction with even good decisions (Haynes, 2009; Reutskaja & Hogarth, 2009), and uncertainty that the selected alternative dominates all other alternatives in the choice set (decision confidence, Chernev, 2003). CO effects have been observed in various situations where consumers are faced with different levels of decision task difficulty, choice set complexity and long-term consequences. Prominent examples comprise grocery shopping (Iyengar & Lepper, 2000), mobile phone connection plan choices (Earl et al., 2019), online dating (Pronk & Denissen, 2020) or health care, e.g. health plan choice (Afendulis et al., 2015; Heiss et al., 2013; Frank & Lamiraud, 2009) and medical treatment (Hafner et al., 2018). Sequential elimination techniques (Besedeš et al., 2015) and the provision of tailored information (Kaufmann et al., 2018) have been proposed to counter CO while keeping freedom of choice.

In recent years, an upcoming debate about more patient involvement in health care (patient empowerment) calls for an intensified participation of (informed) patients and a shared decision making between patients and physicians (Barry & Edgman-Levitan, 2012). Further, increasing patients’ choices of providers has become a dominant focus of health policy promoting (quality) competition among health care providers (García-Lacalle, 2008). Despite stated preferences of individuals for having a choice of health care providers (Schneider-Kamp & Askegaard, 2020; Coulter & Jenkinson, 2005), patients also report that they are overwhelmed by choices and lack the necessary information to make informed choices (Berendsen et al., 2009; Schlesinger, 2010). Hence, it is suggestive to expect the existence of (adverse) CO effects in the demand for health care.

In Germany, patients are free to choose their care provider including free access to specialists without referral from general practitioners (GPs). This form of gatekeeping is obligatory in most other Western countries, e.g. the US, the UK, the Netherlands and Australia (Reibling & Wendt, 2012). Visiting a physician is generally associated with costs due to travel and waiting time (Tur-Sinai & Litwin, 2015). Both are the lower the better is the spatial availability of the demanded health service. However, a generous provision of ambulatory health care services might be characterized by an important asymmetry. On the one hand, a large supply of specialized care increases the choice opportunities of the patients within a region, and might invoke CO effects. For instance, a generous supply of physicians might reduce the patients’ confidence in having selected the dominant specialised treatment from a set of alternative providers (Chernev et al., 2015). This so-called *health uncertainty* emerges from the challenge to choose from an increased set of alternative treatment opportunities with eventually serious aftermath (Han et al., 2011).^1^ On the other hand, a good supply of GPs facilitates the acquisition of care coordination, and might help patients to simplify and improve decision-making by providing tailored information.

In this study, we examine whether there is a CO effect in the demand for ambulatory health care in Germany and, if the coordinating role of the GP providing tailored information is an effective means to improve the patients’ decision quality. More precisely, we estimate the relation between medical service supply and health uncertainty. We expect that the larger the supply of specialists (GPs) the higher (lower) is the health uncertainty. The empirical analysis conditions upon more than 30,000 observations on individual health and health behaviour data from four waves of the German Socio-Economic Panel (SOEP) combined with health care infrastructure data at regional resolution. We apply the ‘within-between’  formulation of Mundlak (1978) to analyze the association between health care infrastructure and health uncertainty.

In the next section, we derive two hypotheses on the association among forms of medical service provision and health uncertainty. Section 3 introduces the data and is explicit on the measure of latent health uncertainty. Moreover, the estimation strategy is illustrated. Empirical results from panel regressions are provided in Sect. 4 and discussed in more detail in Sect. 5. Section 6 summarizes and concludes. Appendix A gives a detailed description of the data and Appendix B summarizes the results of a series of robustness checks.

## Choice overload and the demand for health care

The relationship between physicians and patients is changing towards more patient participation (Barry & Edgman-Levitan, 2012). Being considered as prime providers of health related information, patients may extensively consult physicians in their strive for contributing to informed choices about their health and medical treatment options (Marstedt, 2018). We investigate how the availability of specialized vs. general practice care services might contribute to a CO effect in the demand for ambulatory health care that is expressed in form of health uncertainty. We formulate two hypotheses highlighting associations among the availability of specialized and general practice care, and health uncertainty (H1 and H2).

By virtue of prospect theory a patient’s current health status can be considered as the reference point (Treadwell & Lenert, 1999), and any health benefits that result from a chosen treatment can be interpreted as a gain (Adida, 2021). Therefore, patients face a trade-off when choosing between the available treatment options, since each treatment represents a gain in terms of health benefits and a loss in terms of health benefits that would accrue from disregarded treatments. According to prospect theory, people tend to weight losses more than gains (loss aversion, Kahneman & Tversky, 1979). This has also been observed in the health domain (Attema et al., 2013). As a consequence, patients that compare several treatment options face the risk of a negative net result which increases with the number of considered treatment options. This loss prospect associated with a treatment decision explains a CO effect in the demand for health care that materializes in the form of an increased health uncertainty, i.e. the reduction of a patients’ confidence in having selected the dominant treatment option from a set of alternatives (Chernev et al., 2015).

Gathering information by visiting a physician is generally associated with costs that are mainly due to travel and waiting time (Tur-Sinai & Litwin, 2015). Both depend on the health care infrastructure, i.e. the costs are lower in regions with better spatial availability of the demanded health service. Under free provider choice and direct access to medical specialists (as in Germany; Reibling & Wendt, 2012) individuals can choose from a set that is the larger the better is the spatial availability of the specialised health service. In the context of CO, we formulate our first hypothesis:

- H1: *Health uncertainty is higher in regions with a high density of medical specialists.*

To reduce CO one might think of simply limiting the available choices (Hanoch & Rice, 2006). According to our first hypothesis a reduced choice set should go along with lower health uncertainty.

Under preserving freedom of choice, the economic literature has proposed further approaches to counter CO as, e.g., sequential elimination techniques (Besedeš et al., 2015) and the provision of tailored information (Kaufmann et al., 2018). Both approaches are motivated by theories of limited or selective attention. Accordingly, individuals base their judgements on certain aspects of available options and discard other aspects to cope with information overload (Köszegi & Szeidl, 2012; Kahneman, 1973). For instance, Iyengar and Kamenica (2010) show that people have stronger preferences for simple alternatives that are easy to understand if the size of the choice set grows. The provision of tailored information is supposed to reduce search and comparison costs and, thus, nudges individuals to improve their decision while preserving free choice (Thaler & Sunstein, 2009). In the context of health, Kaufmann et al. (2018) find that the provision of tailored information can help individuals to make better health plan choices. An alternative approach to stimulate optimal decision-making is to reduce a large decision problem into a series of smaller problems (Besedeš et al., 2015). For instance, when breast cancer patients make sequential decisions, they demonstrate greater comprehension of treatment benefits and are able to make better choices as if all decisions are made at once (Zikmund-Fisher et al., 2011).

The coordinating role of GPs comprises sequential elimination techniques and the provision of tailored information. GPs are the first port of call for most patients with a broad variety of health problems (e.g. multiple, complex medical conditions) and focus on continuously treating the whole person through all stages of life (Scott, 2000). By gathering, organizing and arranging the complex medical information for the patient in an understandable and tailored manner, GPs act as knowledge brokers within the patient’s social (physician) network (Burt, 2005). While providing continuous care (typically) over longer time periods, GPs sequentially support their patients in valuing and, thus, reducing the set of alternatives that need to be considered at one time. In this sense, a lasting relationship between a patient and her GP should result in a reduction of health uncertainty. Taking account of patterns of spatial availability as potential moderators of this relation, we formulate the following hypothesis:

- H2: *Health uncertainty is lower in regions with a high density of general practitioners.*

## Data and variables

In this section, we first describe the data sources and the dimensions of our data. Secondly, we outline the estimation of health uncertainty that will serve as the dependent variable. Thirdly, we sketch the ‘within-between’ formulation of the Mundlak approach (Mundlak, 1978) which we employ to examine hypotheses H1 and H2.

### Data sources and panel dimensions

To analyze a potential CO effect in the demand for ambulatory health care we draw data from two sources: the SOEP database for individual health data as well as socio-economic characteristics, and the INKAR database for regional-level information. The SOEP (Schupp et al., 2017) is an annual household panel study. It provides information on individual and household characteristics since 1984 (for further information see Wagner et al., 2007). Starting in 2002, the SOEP covers a set of health related questions every second year, the so-called ‘*SF-12v2*^TM^ Health Survey’ (SF-12, Andersen et al., 2007). We restrict our analysis to individuals aged over 18 who have taken part in at least two waves of the survey between 2006 and 2012.^2^ In total, our data set contains about 30,000 observations for more than 11,000 individuals (see Appendix A for a detailed description of the data set). The INKAR database is managed by the Federal Institute for Research on Building, Urban Affairs and Spatial Development, and provides information on infrastructure and socio-economic characteristics with regional resolution. The geographic scale of our analysis refers to so-called spatial planning units (‘*Raumordnungsregionen*’, ROR).^3^

### Estimation of health uncertainty

To estimate health uncertainty, we build upon ideas of Hubbard et al. (1995) and Palumbo (1999) on measuring health risk with the variance of individual uninsured health care expenditures. However, in countries like Germany with comprehensive health insurance coverage, out-of-pocket expenditures are only of minor economic relevance. Therefore, we follow Jappelli et al. (2007) and consider the perceived health status instead of health care expenditures as the more appropriate variable.

Specifically, we approximate health uncertainty by the estimated variance of the probability of falling into the worst health category. With this purpose we consider an ordered logit regression model for the latent health status $H^{*}_{it}$ of individual *i* in time *t*, i.e.,

1 $$\begin{aligned} H^{*}_{it} = \varvec{w}_{it}\beta + \alpha _i + \varepsilon _{it}, \qquad {\text {for }} i=1,\ldots ,N_t,\; t \in \{2006,2008,2010,2012 \}. \end{aligned}$$

Similar to the analysis in Jappelli et al. (2007), the latent health status $H^{*}_{it}$ in (1) is approximated by the self-reported health status $H_{it}$ (5 = very good, 4 = good, 3 = fair, 2 = poor, 1 = very poor). Random effects $\alpha _i \sim N(0,\sigma _{\alpha }^2)$ capture time invariant unobservable factors that influence an individual’s health status, and $\varepsilon _{it}$ is a random shock to individual health which is assumed to be logistically distributed.^4^ The set of covariates in $\varvec{w}_{it}$ is in line with the approach of Jappelli et al. (2007), and comprises the age ($\texttt{age}$ & $\texttt{age}$^2^), four ordered categories of educational achievements ($\texttt{edu1}$ to $\texttt{edu4}$), year effects and regional dummy variables.^5^ However, in contrast to Jappelli et al. (2007), the household income is not considered due to potential endogeneity.^6^

We run separate ordered logit regressions for female and male respondents. Respective estimation results are documented in Table 1. The results are largely in line with findings of Jappelli et al. (2007). Estimated associations differ slightly between men and women. The estimated negative coefficient of the education (reference: highest level $\texttt{edu4}$) are larger in absolute magnitude for men than for women. For both sexes, we find a convex negative relationship between age and the health status. While the health of men worsens over time (see the significantly negative year effects), it is remarkably stable for women.

**Table 1:** Ordered logit model estimates for self-reported health status

Model	(1)	(2)
Variable	Female	Male
$\texttt{edu1}$	− 1.29***	− 1.89***
$\texttt{edu2}$	− 0.58***	− 1.14***
$\texttt{edu3}$	− 0.44***	− 0.90***
$\texttt{age}$	− 7.90***	− 11.00***
$\texttt{age}$^2^	1.40	4.33***
2008	5.87	− 10.70**
2010	3.03	− 24.40***
2012	0.63	− 16.80***
Observations	17,383	13,339
Individuals	6345	4737

Random effects ordered logit model estimates for self-reported health status $H_{it}$: 5 = very good, 4 = good, 3 = fair, 2 = poor, 1 = very poor. Heteroskedasticity robust standard-errors are used. Significance levels: ***1%; **5%; *10%

Following Jappelli et al. (2007) the estimated health uncertainty statistic is the model implied variance of the probability of the worst health category $P(H_{it}=1)$

2 $$\begin{aligned} HU_{it} = P(H_{it}=1)*(1-P(H_{it}=1)), \quad i=1,\ldots ,N_t,\, t \in \{2006,2008,2010,2012 \}, \end{aligned}$$

where $$P(H_{it}=1)=F(-\varvec{w}_{it}\beta - \alpha _i)$$ and $$F(\varepsilon )=\exp (\varepsilon )/(1+\exp (\varepsilon ))$$.

For an intuitive understanding of $HU_{it}$ as a quantification of health uncertainty it is important to notice that poor health is likely to be one of the most relevant serious adverse events for quality of life (Stucki & Bickenbach, 2019). A high probability of facing very poor health is consistent with low health uncertainty, since people with a high risk ($P(H_{it}=1)$) may be more likely to resign themselves and accept their illness. As a consequence, they are certain about their own health and the variance of the probability is small. Similarly, people having only a minor risk of experiencing very poor health face a low uncertainty about their health. In contrast, a medium health risk goes along with a higher variance. It might evoke fear of deterioration in the health of the healthy, or hope for improvement in the health of the unhealthy. Uncertainty regarding health is heightened for both situations, regardless of whether the risk of experiencing (very) poor health is elevated or diminished.

Descriptive statistics for $H_{it}$, $P(H_{it}=1)$ and $HU_{it}$ are shown in Table 2. The mean health status is 3.08, indicating that on average, individuals evaluate their health as ‘fair’. The assessments vary by approximately one category on average. While the probability of falling into the worst health category is estimated by 4.64%, the respective risk of experiencing very poor health, $HU_{it}$, is a bit smaller, i.e., 3.19%. Both variables are over-dispersed, as their standard deviation exceeds the average outcome. Interestingly and pointing to significant differences between individuals, the between variation is larger than the within variation.

### Empirical analysis

#### The model

To avoid eventual adverse effects of outlying observations on estimation results, panel regressions are performed for $\ln (HU_{it})$ as the dependent variable. For the health uncertainty measure, we implement the ‘within-between’ formulation of the Mundlak approach (Mundlak, 1978). This model takes account of potential correlation between covariates and individual effects, and allows us to differentiate within and between effects (Bell & Jones, 2015). In particular, the latter are of interest to estimate the unspecific associations among the availability of specialized and general practice care (H1 and H2), on the one hand, and health uncertainty, on the other hand. The model reads as

3 $$\begin{aligned} \ln (HU_{it}) = \tilde{\varvec{x}}_{it}\beta ^W + \bar{\varvec{x}}_{i}\beta ^B + \varvec{z}_{i}\gamma ^B + \mu _t + \omega _i + e_{it}, \end{aligned}$$

where $\tilde{\varvec{x}}_{it}$ collects time variant variables that are centered by the individual means, which are stacked in $\bar{\varvec{x}}_{i}$. Here $\beta ^W$ and $\beta ^B$ are vectors consisting of the within and between effects, respectively. Time invariant observable factors are collected in $\varvec{z}_{i}$, $\omega _i \sim N(0,\sigma _{u}^2)$ captures time invariant unobservable heterogeneity, the $\mu _t$ are fixed time effects, and $e_{it}$ is an idiosyncratic error term.

#### Conditioning variables

For the empirical analysis of health uncertainty in (3), we use a set of conditioning variables which is in the tradition of the related literature on modelling health care outcomes (see for instance, Finkelstein et al., 2016; Eibich & Ziebarth, 2014; Herwartz & Schley, 2018; Felder & Tauchmann, 2013). We next describe in some detail covariates measured at the individual and regional level. Descriptive statistics are shown in Table 2.

**Table 2:** Descriptive statistics

Variable	Mean	SD	Between SD	Within SD
Health outcome: $H_{it}$ (5 = very good, 4 = good, 3 = fair, 2 = poor, 1 = very poor)	3.08	0.95	0.82	0.49
Probability of very poor health: $P(H_{it}=1)$ (in %)	4.64	11.10	10.85	0.95
Health uncertainty: $HU_{it}$ (in %)	3.19	5.46	5.37	0.52
*Individual characteristics*				
$\texttt{bmi}$	26.64	4.75	4.59	1.15
$\texttt{smoking}$	0.25	0.44	0.40	0.16
$\mathtt {healthy\_diet}$	0.56	0.50	0.41	0.29
$\texttt{age}$	55.08	16.07	16.17	1.82
$\texttt{male}$	0.43	0.50	0.49	0.00
$\texttt{notGerman}$	0.05	0.21	0.22	0.03
$\texttt{married}$	0.67	0.47	0.45	0.14
$\texttt{edu1}$	0.13	0.34	0.34	0.05
$\texttt{edu2}$	0.50	0.50	0.49	0.08
$\texttt{edu3}$	0.13	0.34	0.33	0.06
$\texttt{edu4}$	0.23	0.42	0.42	0.05
$\texttt{training}$	0.01	0.08	0.06	0.06
$\mathtt {private\_health\_insurance}$	0.14	0.34	0.34	0.07
*Regional variables*				
$\texttt{spec}$	115.80	29.70	28.95	6.53
$\texttt{gp}$	49.37	6.51	6.35	1.45
$\texttt{beds}$	61.68	9.50	9.11	2.69
$\mathtt {spec\_diversity}$	20.88	5.76	5.64	1.31
$\texttt{dialysis}$	5.94	1.65	1.55	0.58
$\mathtt {gdp p.c}$	30.16	8.55	8.37	1.85
$\texttt{education}$	30.19	7.41	6.19	4.18
$\texttt{age65}$	20.61	1.90	1.84	0.46
$\texttt{unemployment}$	9.10	3.95	3.63	1.63
$\texttt{rural}$	0.25	0.44	0.43	0.06
$\mathtt {intermediate\_urban}$	0.30	0.46	0.45	0.06
$\texttt{urban}$	0.45	0.50	0.49	0.06
2006	0.29	0.45	0.18	0.43
2008	0.31	0.46	0.17	0.44
2010	0.28	0.45	0.18	0.42
2012	0.13	0.33	0.17	0.29

The table documents descriptive statistics for 11,082 individuals observed every second year from 2006 to 2012 in the upper panel (30,722 observations), and for the 96 RORs in the lower panel (480 observations). SD: standard deviation

**Individual health behaviour and socio-demographic covariates**

Covariates with highest resolution comprise health related behaviour and socio-demographic characteristics. The former group consists of two dummy variables indicating with values of unity if an individual smokes ($\texttt{smoking}$) or follows a health conscious diet ($\mathtt {healthy\_diet}$). Otherwise these variables take values of zero. The variables capturing socio-demographic characteristics include age ($\texttt{age}$), a set of dummy variables indicating the gender of a person ($\texttt{male}$), nationality ($\texttt{notGerman}$), marital status ($\texttt{married}$) and four ordered categories of educational achievements ($\texttt{edu1}$ to $\texttt{edu4}$),^7^ Further, individuals are indicated whether they are taking part in vocational training, military or community service ($\texttt{training}$), and are privately health insured ($\mathtt {private\_health\_insurance}$).

**Regional characteristics**

**Fig. 1:**
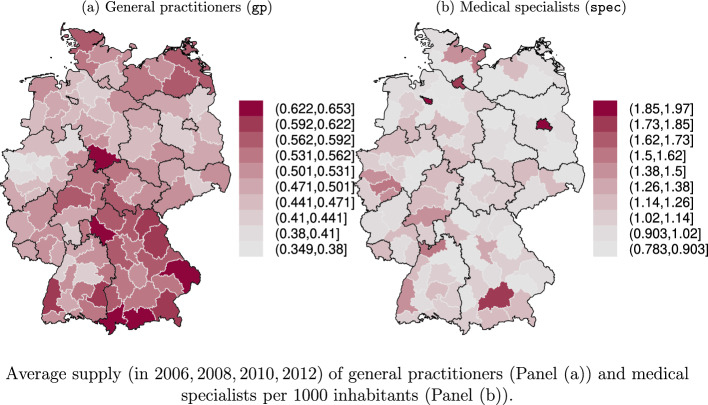
Spatial distribution of health care providers. Average supply (in 2006, 2008, 2010, 2012) of general practitioners (**a**) and medical specialists per 1000 inhabitants (**b**)

Our regression models also condition on the regional provision of health care services (see Sect. 3 for the definition of the level of spatial resolution). In particular, this group of covariates consists of the number of general practitioners ($\texttt{gp}$) and medical specialists ($\texttt{spec}$) (in counts per 1000 inhabitants) which play a central role in testing the hypotheses H1 and H2 developed in Sect. 2. As Fig. 1 illustrates both supply variables show a substantial regional variation. For instance, while Bavaria in the south east of Germany is characterized by a relatively high density of GPs, regions in the western part (North-Rhine Westphalia) face remarkably lower levels of GP supply. The distribution of medical specialists is quite different from the distribution of GPs. Especially the metropolitan regions Berlin, Hamburg, Bremen and Munich show outstandingly high densities of medical specialists. To control for agglomeration effects, we further include dummy variables representing the urbanization level of the region, i.e. $\mathtt {intermediate\_urban}$ and $\texttt{urban}$, such that $\texttt{rural}$ serves as reference.^8^

Further count variables describing the health care infrastructure are the numbers of hospital beds ($\texttt{beds}$) and of dialysis devices ($\texttt{dialysis}$) per 100,000 inhabitants. We consider the latter metric to represent the availability of medical equipment. It is, however, only available at the level of the 16 federal states in Germany.^9^ As in Herwartz and Schley (2018), we use a metric of the heterogeneous provision of specialized outpatient services to account for networking among physicians and risk management across medical specialities ($\mathtt {spec\_diversity}$).^10^ Following Felder and Tauchmann (2013), we complete the set of explanatory variables with regional socio-economic factors, namely the gross domestic product (GDP) per capita ($\mathtt {gdp p.c.}$), the share of matriculated school graduates (‘*Hochschulreife*’) among all school graduates ($\texttt{education}$), the unemployment rate ($\texttt{unemployment}$), and the share of the population older than 65 years ($\texttt{age65}$). The lower panel of Table 2 reports the descriptive statistics for the regional level variables. One can note a considerable variation between regions whereas the within unit variance is smaller in relative terms.

#### Identification

The selected approach is susceptible to criticism that the perceived associations could arise from confounding effects or reverse causality. In fact, an inverse correlation between specialist density and HU as stated in hypothesis H2 might result from the fact that individuals facing high HU may opt to reside in regions with a larger concentration of specialists. Leading to similar correlation pattern, specialised physicians may opt to offer their services in urban areas where they expect a relatively high share of people with medium or high health risk. To address these concerns and enhance the robustness of our empirical strategy, we additionally estimate Model (3) based on data of individuals who have changed their place of living across regions during the sample period. Instead of the between variation, we focus on the within variation by estimating a fixed-effects model. This allows to examine the impact of increased specialist density when an individual relocates to an area with a higher concentration of specialists or vice versa. Similarly, we assess the impact of HU when an individual faces a higher GP density by moving to a corresponding region. To further strengthen the empirical strategy, we include two interaction variables that allow to differentiate the effects of $\texttt{spec}$ and $\texttt{gp}$ for individuals that are moving from less (more) agglomerated to more (less) agglomerated regions. We expect that the estimated interaction effects are insignificant if the associations between $\texttt{spec}$ and $\texttt{gp}$, on the one hand, and HU, on the other hand, are due to the mechanisms postulated in hypotheses H1 and H2.

## Results

In this section, we provide the results from testing a CO effect in the demand for ambulatory health care in Germany, and evaluate the role of the health care infrastructure in shaping individual health uncertainty. We analyze the conditional properties of estimated health uncertaintiy ($HU_{it}$) and test the hypotheses H1 and H2 (Table 3). Further, we apply a series of robustness checks. The respective results are provided in Appendix B.

The results of the regression analysis of health uncertainty are shown in Table 3. The applied Mundlak approach separates within and between effects of time varying variables.

**Table 3:** Determinants of Health Uncertainty

Model	(3)	
Variable	$\beta ^W$	$\beta ^B$
	Within	Between
Individual characteristics		
*Health-related behaviour*		
$\texttt{bmi}$/100^a^	0.13**	6.91***
$\texttt{smoking}$/10^a^	0.07*	5.54***
$\mathtt {health\_diet}$/10^a^	0.01	− 2.29***
*Socio-Demographic factors*		
$\texttt{age}$/10^a^	1.34***	0.59***
$\texttt{male}$		− 0.33***
$\texttt{notGerman}$		− 0.02
$\texttt{married}$	0.03***	− 0.04
$\texttt{edu1}$	1.26***	1.07***
$\texttt{edu2}$	0.66***	0.58***
$\texttt{edu3}$	0.53***	0.52***
$\texttt{training}$	0.02	0.12
$\mathtt {private\_health\_insurance}$	0.00	− 0.20***
Regional variables		
*Medical infrastructure*		
$\texttt{spec}$	0.03	0.19**
$\texttt{gp}$	0.08	− 1.71***
$\texttt{beds}$	− 0.04	0.24
$\mathtt {spec\_diversity}$	0.02	1.37**
$\texttt{dialysis}$/10^a^	0.07*	0.12
*Demographic and socio-economic characteristics*		
$\mathtt {ln(gdp p.c)}$	− 0.57***	− 0.51***
$\texttt{education}$/100^a^	− 0.07***	− 1.66***
$\texttt{unemployment}$/100^a^	− 0.36	0.49
$\texttt{age65}$/100^a^	− 0.90	− 4.40***
*Regional Urbanization*		
$\mathtt {intermediate\_urban}$		0.05
$\texttt{urban}$		0.04
Time effects		
2008	− 0.11***	
2010	− 0.19**	
2012	− 0.32**	
Intercept	− 7.15***	
Number of observations	30722	
Number of individuals	11082	

Estimated by means of the ‘within-between’ formulation of the Mundlak approach (3)

^a^Variables are transformed for ease of display. Heteroskedasticity robust standard-errors are used. Significance levels: ***1%; **5%; *10%

The estimated regression coefficient for the between effect of the number of medical specialists ($\texttt{spec}$) is significantly positive. This result is in line with hypothesis H1. Opportunities to choose from a rich set of medical service providers reduce the costs for gathering information by visiting a physician (Tur-Sinai & Litwin, 2015) and, thus, increase the considered number of treatment options. Patients in regions with a large supply of medical specialists might face the risk of a negative net result when comparing too many treatment options. Hence, suggesting the existence of a CO effect in the demand for health care, the (health) uncertainty about having selected the dominant treatment option from a set of alternatives is the stronger the larger is the supply of specialists (Chernev et al., 2015).

Moreover, our results further suggest that the CO effect is reduced in regions featuring a generous supply of general practice services. Accordingly, regions with a larger supply of GPs ($\texttt{gp}$) face lower levels of health uncertainty. This result supports hypothesis H2. Reduced costs of visiting a GP (Tur-Sinai & Litwin, 2015) increase the probability to first visit a GP in case of a health problem. At the patient side, the search and comparison costs of treatment decisions are reduced through tailored information provided by the GP, thereby nudging individuals to improve their decision while preserving free choice of treatment options (Thaler & Sunstein, 2009). Charles et al. (1997) note that many medical treatments involve more than one physician in the decision making process each with a specific treatment preference. In such situations, GPs may provide guidance for patients and help them to reduce or structure the set of alternative treatments. Optimal decision-making is stimulated by reducing a large decision problem into a series of smaller problems (Besedeš et al., 2015).

In Table 4, the estimated within effects of $\texttt{spec}$ and $\texttt{gp}$ are shown based on 1121 observations of 384 individuals that moved across regions during the sample period. The estimates are very similar to the between effects of the model based on the full sample. Further, the interaction effects that differentiate the effects of $\texttt{spec}$ and $\texttt{gp}$ between individuals moving from regions with different agglomeration levels are insignificant. These findings support the view that the estimated associations of $\texttt{spec}$ and HU as well as $\texttt{gp}$ and HU are due to the postulated mechanisms in hypotheses H1 and H2, respectively.

**Table 4:** Fixed effects estimation for mover only

Model	(4)	(5)
Variable	$\beta ^W$	$\beta ^W$
	Within	Within
*Medical infrastructure*		
$\texttt{spec}$	− 1.84***	− 1.96***
$\texttt{gp}$	0.22**	0.24**
*Interactions*		
$\texttt{spec}$ $\times$ $\texttt{rural2urban}$		− 0.22
$\texttt{spec}$ $\times$ $\texttt{urban2rural}$		0.17
$\texttt{gp}$ $\times$ $\texttt{rural2urban}$		2.19
$\texttt{gp}$ $\times$ $\texttt{urban2rural}$		2.87
Number of observations	1121	1121
Number of individuals	384	384

Estimated by fixed-effects. $\texttt{rural2urban}$/$\texttt{urban2rural}$ are dummy variables identifying individuals who have moved from less (rural &intermediate urban)/more (intermediate urban &urban) agglomerated to more (intermediate urban &urban)/less (rural &intermediate urban) agglomerated regions. Interaction terms are generated by multiplying $\texttt{rural2urban}$ and $\texttt{urban2rural}$ with the centered density of GPs and specialists, respectively. Heteroskedasticity robust standard-errors are used. Other control variables are the same as those used for the estimation in Table 3, but the respective estimates are not shown due to space considerations. Heteroskedasticity robust standard-errors are used. Significance levels: ***1%; **5%; *10%

## Discussion

The results of this study suggest a CO effect in the demand for ambulatory health care in Germany that is expressed by higher health uncertainty for people facing more treatment opportunities. More specifically, our results highlight (1) that patients who live in an area with a large supply of specialists are subject to CO effects, and (2) that CO effects are reduced in regions featuring a generous supply of general practice services. The latter result suggests that the coordinating role of GPs, comprising sequential elimination of treatment options and the provision of tailored information, seems to be effective to reduce the CO effect, while preserving free choice.

Similar to consumers at other markets, patients seem to be overwhelmed to choose the best option when they are confronted with medical treatment decisions. From a health policy perspective, this challenges the effectiveness of freedom of choice as a means to improve health care services (Vrangbaek et al., 2012; García-Lacalle, 2008). Consumer choice is expected to promote competition among health care providers making them to provide health care with higher quality and more efficiently (Propper et al., 2006). As shown by Huck et al. (2016) in a laboratory experiment, competition in form of free choice of physicians reduces (inefficient) overtreatment. To increase the ability of patients to choose the best option or provider, recent legislative efforts in several countries have advanced public reporting on the quality of care (Kumpunen et al., 2014). However, the impact on quality of care has been small (Campanella et al., 2016; Strumann et al., 2022) due to a rather low mobility of patients to obtain the best quality of care (Avdic et al., 2019; Moscelli et al., 2016). Although, these findings consider hospital care, our results might suggest that more information and a larger set of providers to choose from will not automatically lead to an improved quality of patients’ decisions. Instead, patients seeking information from public reporting might also face the risk of a negative net result when comparing too many providers and choose the nearest provider to simplify decision making.

However, the quality of patients’ decisions might be improved by an increased coordination of the GPs. Patients want to have the free choice of health care providers (Schneider-Kamp & Askegaard, 2020; Coulter & Jenkinson, 2005), but are overwhelmed by treatment opportunities and lack the necessary information to make informed choices (Berendsen et al., 2009; Schlesinger, 2010). Moreover, potential CO effects can easily aggravate noticing that the choices under scrutiny are subject to the challenge to choose from a set of alternative treatment opportunities with eventually serious aftermath under uncertainty (Han et al., 2011; McNeil, 2001). Meanwhile the internet facilitates information-seeking behaviours (Weaver et al., 2010), and also increases the amount of information to be processed by the patient. In contrast to specialists that are trained to treat specific illnesses, the focus of the GPs’ medical education is the continuous treatment of the whole person through all stages of life with a broad variety of (potential) health problems (Scott, 2000). Therefore, they should be able to organize and arrange complex medical information for the patient in an understandable and tailored manner. The provision of personalized information reduces the set of options that need to be compared and, thus, reduces the risk of a negative net result when comparing too many options. As shown by Kaufmann et al. (2018), providing personalized information can effectively support individuals in making better health plan choices. The provision of personalized information seems to increase the awareness of the choice set and also reduces decision times.

The continuous care typically provided over extended periods of time enables GPs to persistently support their patients in valuing treatment alternatives and, thus, split the set of options that need to be considered at one time into a series of smaller ones. In this regard, findings of Besedeš et al. (2015) suggest, however, that such a sequential elimination strategy can be subject to a status quo bias, i.e., the most recent selection is overvalued in the next decision. This form of inertia might also be relevant for medical decisions, especially when a patient visits sequentially several specialists and undervalues new treatment options. Besedeš et al. (2015) further show that the most beneficial decisions are made under a sequential architecture that integrates the previously selected options into a final set from which an ultimate decision is made. Similarly, Chernev and Hamilton (2009) observe a preference of consumers for smaller assortments, if these include the most attractive options from larger choice sets. A GP that coordinates and delegates the continuous care of the patient by treating and, if necessary, referring the patient to medical specialists gathers, organizes and arranges the complex medical information for the patient to guide the decision making. This type of knowledge brokering provides personalized information in a sequential elimination architecture as proposed by Besedeš et al. (2015). Therefore, firstly visiting a GP should enable patients to make their treatment decisions with less effort and, thus, avoid short-cuts to oversimplify decision making. Such oversimplifications or poor decisions can have adverse consequences on overall health channeled through the demanding of avoidable medical treatments or delaying or even dropping the decision process (Khaleel et al., 2020).

## Conclusion

Patient empowerment calls for an intensified participation of (informed) patients with rising opportunities to choose freely among providers and treatments leading to an increased responsibility of the patient for her medical care. The informed patient should be able to oppose perceived physician paternalism and promote competition among health care providers making them to supply health care with higher quality and in an efficient manner. However, similar to other consumer markets, our study shows that having more choice opportunities can also have negative consequences for patients.

Our results suggest a choice overload effect in the demand for ambulatory health care in Germany. Patients that are confronted with a large choice set of available treatment alternatives and physicians have a reduced confidence in having selected the dominant specialised treatment from the set of alternative providers. The coordinating role of the GP has the scope to guide patients through the decision making process and to reduce the patients’ health uncertainty.

## Supplementary Information

Below is the link to the electronic supplementary material.

Supplementary file 1 (pdf 50 KB)

## Footnotes

1 The medical literature has well established the role of patient uncertainty for shared decision making by physicians and patients. Moreover, issues related to health uncertainty are part of the education of medical students (Hall, 2002).

2 From 2004 until 2012 a quarterly copayment for ambulatory care in Germany had to be paid by individuals with statutory health insurance (SHI) who account for about 86% of the population. To immunize the analysis against adjustment effects, we exclude the years 2004 and 2014 from the sample.

3 The RORs are artificially created for statistical purposes. Germany consists of 96 RORs which are similar to the European NUTS-2 regions (‘*Nomenclature des unités territoriales statistiques*’).

4 In preliminary analysis, we have also considered ordered probit specifications. However, the logit model showed computational merits and, unlike the probit model, it did not suffer from any convergence failures.

5 The categories ($\texttt{edu1}$ $\texttt{edu2}$) characterize individuals with an educational achievement of elementary and (lower) secondary education (ISCED, International Standard Classification of Education 2011). The third category ($\texttt{edu3}$) consists of individuals with vocational training and Bachelor degree. The last category ($\texttt{edu4}$) refers to individuals with (higher) academic degrees (Master degree, PhD or equivalent).

6 If we include the logarithm of the monthly household income (adjusted for purchasing power) in the ordered logit regression model, the results remain very similar.

7 The categories ($\texttt{edu1}$ $\texttt{edu2}$) characterize individuals with an educational achievement of elementary and (lower) secondary education (ISCED, International Standard Classification of Education 2011). The third category ($\texttt{edu3}$) consists of individuals with vocational training and Bachelor degree. The last category ($\texttt{edu4}$) refers to individuals with (higher) academic degrees (Master degree, PhD or equivalent).

8 Alternatively, we considered the population density as a control variable. The model fit resulting from this robustness analysis is somewhat weaker in comparison with the benchmark indicators. The estimated coefficients of the remaining variables are very similar.

9 The number of regional units (RORs) varies across federal states between one (Berlin, Bremen, Hamburg) and 19 (Baden-Wuerttemberg).

10 We take the standard deviation within each region across count statistics for five medical specialist groups (internists, ophthamologists, orthopaedistis, and psychotherapists per 1000 inhabitants and paediatricians per 1000 children.).
