# Supplementary material for: Too many cooks could spoil the broth: choice overload and the provision of ambulatory health care
Source: Int J Health Econ Manag. 2024 May 27;24(3):357–73. doi: 10.1007/s10754-024-09379-y (PMC11445302; doi:10.1007/s10754-024-09379-y)
Supplement: Supplementary file 1 — (pdf 50 KB) [file 10754_2024_9379_MOESM1_ESM.pdf]

Online-Appendix

A Data Description

A.1 Individual Data

Individual data are drawn from the SOEP database (SOEP V.31) in a panel long format (wave 31l). In particular, variable extraction relied on the databases *hgen*, *hl*, *pl*, *ppfadl*, *pequiv*, *pgen*, *health*, *kreise_l* and *ror_long*. Table 5 provides a listing of variables.

Table 5: Variable Summary

Variable	SOEP Variable	description
Health outcome		
H_{it}	gh_nbs	How would you describe your current state of health? (5=very good, 4=good, 3=fair, 2=poor, 1=very poor)
Health related variables		
healthy_diet	plb0095	healthy_diet = 1 if ple0095 = 1 of ple0095 = 2
smoking	pce0005	smoking = 1 if ple0095 = 1
Socio-demographic variables		
age	syear, gebjahr	syear − gebjahr
married	d11104	married = 1 if d11104 = 1
male	d1110211	male = 1 if d1110211 = 1
nationality	pgnation	nationality = 1 if pgnation $\neq$ 1
edu1	pgisced11	edu1 = 1 if pgisced11 $\leq$ 2
edu2	pgisced11	edu2 = 1 if pgisced11 = 3
edu3	pgisced11	edu3 = 1 if 4 $\leq$ pgisced11 $\geq$ 6
edu4	pgisced11	edu4 = 1 if pgisced11 $\geq$ 7
training	plb0022	training = 1 if plb0022 = 3, plb0022 = 16 or plb0022 = 7
health_insurance	ple0097	health_insurance = 1 if ple0097 = 2

The first columns gives the variable used in the analysis. The second and third column show the variable as given in the SOEP data set and a description of its calculation.

A.2 Regional Variables

Data on **health care infrastructure** with regional resolution are drawn from the INKAR database. It provides annual data on the number of physicians and GPs which rely on original

statistics of the National Association of Statutory Health Insurance Physicians (*'Ärzteregister der Kassenärztlichen Bundesvereinigung'*). Data on 'independent ' physicians is not available at the regional level such that we base our analysis on 'publicly funded' physicians. Counts for medical specialists are obtained as the total number of physicians minus the number of GPs. Information on hospital beds and dialysis devices is also available at INKAR and rely on original hospital statistics (*'Krankenhausstatistik des Bundes und der Länder'*).

The INKAR database also comprises **socio-economic and demographic characteristics**. In particular we sample the gross domestic product per capita in 1000 Euro. Education is the share of school graduates with matriculation standard (*'Hochschulreife'*) in all school leavers. As defined by the Federal Employment Agency (*'Bundesagentur für Arbeit'*) the unemployment rate is the share of unemployed in the labour force. The share of the elderly is the fraction of the population aged 65 or older.

B Robustness Checks

In this appendix, we tested robustness of results in subgroups defined by gender, age, education and health status. We divided the sample in men and women, persons aged less or equal 65 years and above 65, persons that have achieved a lower (`edu1`, `edu2`) or higher educational level (`edu3`, `edu4`), and individuals that are sick, medium healthy and healthy. Persons are identified as sick if their sf12 score is within the 1st tercile ($< 33\%$) of the sample. Accordingly, we consider individuals as healthy, if their SF12 score is within the 3rd tercile ($> 66\%$). Medium healthy people have a score which is within the 2nd tercile ($33\% - 66\%$).

Table 6 shows the estimated between effects (β^B) of `gp` and `spec`. In general, the vast majority of estimated effects are in line with with both hypotheses H1 and H2. However, there are some differences between the groups that might provide further insights. For instance, the medical infrastructure of GPs and specialists seem to have higher impacts on the health uncertainty for women than for men. Moreover, stronger effects are observed for individuals that are aged below 65, have higher education, or are healthier.

Table 6: Subgroup Regression Results

Model	observations	individuals	`spec`	`gp`
baseline model	30722	11082	0.19**	−1.71**
female only	17383	6345	0.30**	−2.87**
males only	13339	4737	0.07	0.49
aged under 65	20632	7948	0.23**	−2.11**
aged over 65	10090	3948	0.30	2.21*
lower education	19501	7124	0.06	−1.36**
higher education	11221	4171	0.32**	−2.39**
sick (sf12-1st terc)	10240	5368	−0.16	0.22
med-healthy (sf12-2nd terc)	10241	6385	−0.02	−1.51
healthy (sf12-3rd terc)	10241	5598	0.38**	−3.17**

Within effects of `gp` and `spec` on health uncertainty estimated by means of the 'within-between' formulation of the Mundlak approach (3). To economize on space the coefficients of other the covariables are not shown here. Heteroskedasticity robust standard-errors are used. Significance levels: *** 1%; ** 5%; * 10%.
